# Regulatory role of regucalcin in heart calcium signaling: Insight into cardiac failure (Review)

**DOI:** 10.3892/br.2014.245

**Published:** 2014-03-05

**Authors:** MASAYOSHI YAMAGUCHI

**Affiliations:** Department of Hematology and Medical Oncology, Emory University School of Medicine, Atlanta, GA 30322, USA

**Keywords:** regucalcin, Ca^2+^ signaling, sarcoplasmic reticulum Ca^2+^-ATPase, Ca^2+^-ATPase, nitric oxide synthase, heart failure, superoxide dismutase

## Abstract

Regucalcin was first identified in 1978 as a regulatory protein of Ca^2+^ signaling in liver cells. Regucalcin was shown to play a multifunctional role in cell regulation, such as maintainance of intracellular Ca^2+^ homeostasis and suppression of signal transduction, protein synthesis, nuclear function, cell proliferation and apoptosis in various types of cells and tissues. Cardiac excitation-contraction coupling is based on the regulation of intracellular Ca^2+^ concentration by the Ca^2+^ pump in the sarcoplasmic reticulum of heart muscle cells. Regucalcin, which is expressed in the heart, was found to increase rat heart sarcoplasmic reticulum Ca^2+^-ATPase activity and ATP-dependent Ca^2+^ uptake and mitochondrial Ca^2+^-ATPase activity. Regucalcin was also shown to suppress Ca^2+^-dependent protein tyrosine phosphatase, Ca^2+^/calmodulin-dependent protein phosphatase (calcineurin) and nitric oxide (NO) synthase activity in the heart cytoplasm. Moreover, regucalcin was found to activate superoxide dismutase (SOD), which plays a significant role in the prevention of cell death and apoptosis in the heart. Regucalcin may be a key molecule in heart muscle cell regulation through Ca^2+^ signaling. Regucalcin may also play a pathophysiological role in heart failure. The aim of this study was to review the recent findings regarding the role of regucalcin in Ca^2+^ signaling in the heart.

## 1. Introduction

Regucalcin was first identified in 1978 as a Ca^2+^-binding protein that does not contain an EF-hand motif as a Ca^2+^-binding domain, which is present in numerous Ca^2+^-binding proteins (1). The name ‘regucalcin’ was proposed for this protein, which was shown to suppress Ca^2+^-dependent activation of various enzymes in liver cells (1–4). The regucalcin gene is localized on the X chromosome (5,6). Regucalcin was identified in >15 species and is highly conserved in vertebrate species (7,8). The expression of regucalcin mRNA and protein is regulated through various hormonal stimuli and physiological conditions (7–9).

Regucalcin was shown to play a multifunctional role in cell regulation in the liver and kidney (5,10–13). Regucalcin plays a pivotal role in maintaining intracellular Ca^2+^ homeostasis and suppressing signal transduction, nuclear Ca^2+^-dependent protein kinase and protein phosphatase activity, Ca^2+^-activated deoxyribonucleic acid (DNA) fragmentation and DNA and ribonucleic acid synthesis. Regucalcin was also shown to suppress protein synthesis and activate proteolysis, suggesting a role in protein turnover. The overexpression of endogenous regucalcin was demonstrated to suppress cell proliferation (14) and apoptosis (15), which is mediated through various signal transduction pathways, in various types of cells. Moreover, regucalcin was shown to regulate the gene expression for a number of proteins, suggesting a role as a novel transcription factor (16).

**Figure 1:**
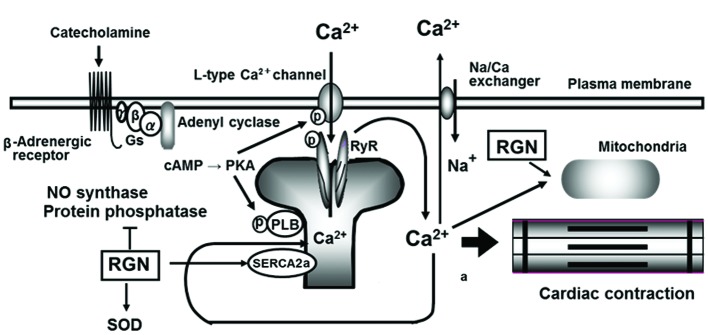
Role of regucalcin (RGN) in the regulation of heart cell Ca^2+^ signaling. Regucalcin is expressed in heart cells. Regucalcin stimulates Ca^2+^ uptake in the sarcoendoplasmic reticulum and mitochondria through activating the sarcoendoplasmic reticulum Ca^2+^-ATPase (SERCA2a) and the mitochondrial Ca^2+^-ATPase, which are Ca^2+^ pump enzymes maintaining intracellular Ca^2+^ homeostasis. Regucalcin suppresses Ca^2+^/calmodulin-dependent protein phosphatase and nitric oxide (NO) synthase activity and activates superoxide dismutase (SOD) in the heart cytoplasm. cAMP, cyclic AMP; PKA, protein kinase A; P, phosphorylation; RyR, ryanodine receptor; PLB, phospholanbane.

Moreover, there is growing evidence that regucalcin is involved in the regulation of heart cell function. The Ca^2+^ current is one of the most important components in cardiac excitation-contraction coupling. This coupling mechanism is based on the regulation of intracellular Ca^2+^ concentration by the Ca^2+^ pump in the sarcoplasmic reticulum of heart muscle cells (17–19). Regucalcin is expressed in the heart and was found to increase rat heart sarcoplasmic reticulum Ca^2+^-ATPase (SERCA2a) activity and ATP-dependent Ca^2+^ uptake and mitochondrial Ca^2+^-ATPase activity (20,21). Regucalcin was also shown to regulate the activities of various enzymes, which are associated with Ca^2+^ signaling in the heart cytoplasm. Therefore, regucalcin may be a key molecule in heart muscle cell regulation. The aim of this review was to discuss the regulatory role of regucalcin in heart Ca^2+^ signaling (Fig. 1), with insight into cardiac failure.

## 2. Expression of regucalcin in the heart

The expression of regucalcin in rat hearts was initially demonstrated by immunohistocemical analysis (22). Regucalcin mRNA is expressed in rat heart muscle and regucalcin is present in the cytoplasm but not the microsomes of rat heart cells (20,23). Regucalcin concentration in the heart muscle tissues was estimated to be ~3.86×10^−8^ M (23). Regucalcin gene expression may be enhanced through various transcriptional factors. Nuclear factor I-A1 (NFI-A1), a transcription factor, was found to be expressed in rat hearts (24) and was shown to specifically bind to the TTGGC motif in the regucalcin gene promoter region (24). In addition, RGPR-p117, a novel transcription factor that binds to the regucalcin gene promoter region (25), was found to be expressed in rat hearts (9,25).

Regucalcin mRNA expression may be altered under various pathophysiological conditions. It was previously demonstrated that regucalcin mRNA and protein levels in the hearts of male and female rats decreased with increasing age, as they were found to be lower in 50-week-old compared to those in 5-week-old rats (26). The effect of 1,1-diphenyl-2-picrylhydrazyl (DPPH), a compound that produces free radicals, on regucalcin mRNA expression in the hearts of 5-week-old female rats was previously investigated (26). Heart regucalcin mRNA levels were found to be reduced at 60 or 180 min following a single intraperitoneal administration of DPPH (5 mg/kg body weight), suggesting that free radical stress exerts a suppressive effect on gene expression. DPPH is potently toxic and normal (wild-type) female rats died within ~300 min following a single intraperitoneal administration of DPPH (5 mg/kg body weight), whereas regucalcin transgenic (TG) female rats died within ~150 minutes after the administration (25). The heart content of regucalcin protein in DPPH-administered rats was shown to be higher in regucalcin TG compared to that in wild-type rats (25). The death of regucalcin TG rats may be accelerated following the administration of free radical-generating compounds. The overexpression of endogenous regucalcin may not contribute to the suppression of free radical stress, as regucalcin was not found to be a free radical scavenger in rats.

The presence of regucalcin in normal vs. dystrophic fibres was demonstrated using comparative mass spectrometry-based proteomics screening (27). Following separation by two-dimensional gel electrophoresis, the spot pattern of the normal vs. the X-linked muscular dystrophy (mdx) diaphragm muscle proteome was evaluated by densitometry (27). The expression levels of 20 major protein spots were shown to be altered and their identity was determined by mass spectrometry (27). A 2-fold reduction of regucalcin in the mdx diaphragm, as well as in dystrophic limb and heart muscle, was confirmed by immunoblotting in young as well as aged mdx mice (27). The results from the proteomics analysis of the dystrophic diaphragm support the hypothesis that abnormal Ca^2+^-handling is involved in X-linked muscular dystrophy (27). A decrease in regucalcin levels may be implicated in insufficient maintenance of Ca^2+^ homeostasis and dysregulation of Ca^2+^-dependent enzymes, resulting in the disturbed intracellular signaling mechanisms that characterize dystrophinopathies (27).

## 3. Regucalcin regulates intracellular Ca^2+^ homeostasis in the heart

The mechanism of cardiac excitation-contraction coupling is based on the regulation of intracellular Ca^2+^ concentration by the Ca^2+^ pump in the sarcoplasmic reticulum of heart muscle cells (17–19). The regulatory effect of regucalcin on Ca^2+^ pump activity in the microsomes (sarcoplasmic reticulum) of rat heart muscle was previously investigated (20). The activity of SERCA2a was found to be increased in the presence of regucalcin (10^−10^-10^−8^ M) at physiological levels in the enzyme reaction mixture (20). However, this increase was not observed in the presence of thapsigargin (10^−5^ M), a specific inhibitor of the microsomal Ca^2+^-ATPase (28), indicating that regucalcin activates Ca^2+^-ATPase in the sarcoplasmic reticulum.

Regucalcin (10^−10^-10^−8^ M) was shown to stimulate ATP-dependent ^45^Ca^2+^ uptake by the microsomes (20). The stimulatory effect of regucalcin on SERCA2a activity was completely inhibited in the presence of digitonin, which exerts a solubilizing effect on membranous lipid, or *N*-ethylmaleimide (NEM), a modifying reagent of sulfhydryl (SH) groups (20). Dithiothreitol (DTT), a protecting reagent of SH groups, markedly increased Ca^2+^-ATPase activity. In the presence of DTT (5 mmol/l), regucalcin was not able to enhance SERCA2a activity (20). The abovementioned findings suggest that regucalcin binds to the lipids at the site close to the Ca^2+^-ATPase in the heart microsomes, acts on the SH groups, which may be the active site of the enzyme, and stimulates Ca^2+^-dependent phosphorylation of the Ca^2+^-ATPase (20). The stimulatory effect of regucalcin on Ca^2+^-ATPase activity was completely inhibited following the addition of vanadate (1 mmol/l), an inhibitor of enzyme phosphorylation (20). In addition, the effect of regucalcin on Ca^2+^-ATPase activity was not modulated in the presence of dibutyryl cyclic AMP (cAMP), inositol 1,4,5-trisphosphate, or calmodulin, which is an intracellular signaling factor (20). Thus, regucalcin was found to increase Ca^2+^-ATPase activity and ATP-dependent Ca^2+^ uptake in rat heart microsomes, which regulates intracellular Ca^2+^ concentration during cardiac excitation-contraction coupling, suggesting a pivotal role for regucalcin in the regulation of heart muscle function.

Phospholamban was shown to regulate SERCA2a activity in heart muscle (29). Ca^2+^-ATPase is activated through cAMP-dependent phosphorylation of phospholamban following hormonal stimulation. The function of the endogenous activator protein of SERCA2a has not been clearly determined. Regucalcin, which is present in the cytoplasm of heart muscle cells, may play an important role as an endogenous activator in the regulation of SERCA2a activity in rat heart muscle (20). Regucalcin may also play a physiological role in the regulation of cardiac excitation-contraction coupling.

Augmentation of regucalcin in regucalcin TG male rats was shown to enhance SERCA2a activity in the heart (30). Western blot analysis revealed a significant increase of regucalcin protein in the cytoplasm of regucalcin TG female rat heart cells, compared to that in wild-type female rats (30). Heart muscle SERCA2a activity was enhanced in TG rats *in vivo* and the changes in the enzyme activity in TG rats were completely abolished in the presence of anti-regucalcin monoclonal antibody (100 ng/ml) in the enzyme reaction mixture (30). Thus, endogenous regucalcin plays a role as an activator in the regulation of heart SERCA2a.

The role of regucalcin in the regulation of Ca^2+^-ATPase activity in rat heart mitochondria was also demonstrated (21). The mitochondrial Ca^2+^-ATPase activity was increased with increasing concentrations of CaCl_2_ (2.5–50 μM) (21). An increase in the enzyme activity was saturated at 50 μM CaCl_2_. The addition of regucalcin (10^−11^-10^−8^ M) to the enzyme reaction mixture led to an increase in Ca^2+^-ATPase activity in heart mitochondria in the presence of 50 μM CaCl_2_ (21). Regucalcin exerted no effects on mitochondrial Mg^2+^-ATPase activity. Furthermore, regucalcin did not exert a significant effect on Ca^2+^-ATPase activity in the presence of digitonin, which was shown to solubilize membranous lipids (21). The stimulatory effect of regucalcin on mitochondrial Ca^2+^-ATPase activity was not observed in the presence of ruthenium red or lanthanum chloride, which are inhibitors of the mitochondrial Ca^2+^ uniporter (21). The stimulatory effect of regucalcin on mitochondrial Ca^2+^-ATPase activity was not observed in the presence of calmodulin or dibutyryl cAMP, which is an intracellular signaling factor that causes an increase in enzyme activity (21). Of note, mitochondrial regucalcin localization was found to be increased in the hearts of regucalcin TG rats compared to that in wild-type rats, as determined by western blot analysis. Ca^2+^-ATPase activity was also increased in the heart mitochondria of regucalcin TG rats (21). The abovementioned findings demonstrate that regucalcin exerts an activating effect on Ca^2+^-ATPase in rat heart mitochondria.

Regucalcin was previously shown to reduce agonist (histamine)-induced Ca^2+^ transients in regucalcin-transfected COS-7 cells and increase their Ca^2+^ storage capacity (31). These observations may be explained by the increased mRNA and protein expression levels of SERCA2a in regucalcin-transfected cells (31). Therefore, the downregulation of regucalcin expression may contribute to the characteristics of disturbed regulation of age-dependent Ca^2+^ homeostasis by decreasing SERCA2a levels (31).

## 4. Regucalcin regulates Ca^2+^ signaling-dependent enzyme activity

Protein phosphorylation-dephosphorylation is a universal mechanism by which numerous cellular events are regulated (32). There are a number of phosphatases that, similar to kinases, are elaborately and rigorously controlled (32). Protein phosphatases are involved in intracellular signal transduction due to hormonal stimulation. Ca^2+^/calmodulin-dependent protein phosphatase (calcineurin), a calmodulin-binding protein, was shown to possess a Ca^2+^-dependent and calmodulin-stimulated protein phosphatase activity (33,34). Cardiac hypertrophy is induced by calcineurin, which dephosphorylates the transcription factor NF-A3, enabling it to translocate to the nucleus (34). In addition, TG mice, which express activated forms of calcineurin or NF-AT3 in the heart, may develop cardiac hypertrophy and heart failure that mimic human heart disease (34), suggesting the existence of a novel hypertrophic signaling pathway. Thus, protein phosphatases play an important role in intracellular signal transduction due to hormonal stimulation in heart cells.

The role of regucalcin in the regulation of protein phosphatase activity in the heart muscle cytosol was demonstrated using regucalcin TG rats (35). Protein phosphatase activity was assayed in a reaction mixture containing the cytosolic protein in the presence of phosphotyrosine, phosphoserine and phosphothreonine (35). The addition of CaCl_2_ (10 and 20 μM) to the enzyme reaction mixture led to an increase in protein phosphatase activity towards three phosphoaminoacids (35). This increase was enhanced following the addition of calmodulin. The addition of regucalcin (10^−9^ and 10^−8^ M) was found to inhibit protein phosphatase activity towards three phosphoaminoacids in the presence of ethylene glycol-bis(2-amino-ethyl)-N,N,N′,N′-tetraacetic acid (EGTA) (35). The inhibitory effect of regucalcin was also observed in the presence or absence of CaCl_2_ (10 μM). Thus, regucalcin was found to inhibit the activity of various protein phosphatasees, dependently or independently of Ca^2+^.

Regucalcin TG female rats were shown to markedly express endogenous regucalcin protein in the heart cytoplasm compared to wild-type female rats (35). Protein phosphatase activity towards three phosphoaminoacids was significantly decreased in the heart cytoplasm of TG rats (35). The effect of Ca^2+^ addition on increasing protein phosphatase activity towards three phosphoaminoacids was not observed in the heart cytoplasm of TG rats (35), supporting the hypothesis that endogenous regucalcin plays a suppressive role in the regulation of protein phosphatase activity in rat heart cytoplasm. Thus, regucalcin was shown to suppress Ca^2+^-dependent protein tyrosine phosphatase and calcineurin activity in the heart cytoplasm of rats (35). The overexpression of regucalcin, which exerts suppressive effects on calcineurin activity, may play a pathophysiological role in the prevention of the development of cardiac hypertrophy and heart failure.

The role of regucalcin in the regulation of protein kinases in the heart remains to be elucidated. Regucalcin was shown to suppress Ca^2+^/calmodulin-dependent protein kinase and protein kinase C in the liver, kidney and brain (12–14).

Moreover, it was demonstrated that regucalcin plays a role in the regulation of nitric oxide (NO) synthase activity in the cytosol of rat heart muscle (36). The addition of CaCl_2_ (5–20 μM) to the enzyme reaction mixture containing the heart cytosolic protein led to an increase in NO synthase activity (36). The Ca^2+^ effect was inhibited by trifluoperazine (TFP), an antagonist of calmodulin, indicating the presence of Ca^2+^/calmodulin-dependent NO synthase activity in rat heart muscle cytosol (36). The activity of NO synthase was decreased following the addition of regucalcin (10^−9^ or 10^−8^ M) (36). This effect was also observed in the presence of CaCl_2_, TFP or EGTA, a chelator of Ca^2+^. The downregulating effect of regucalcin on NO synthase activity was not observed in the presence of *N*^ω^-nitro-L-arginine methyl ester, an inhibitor of the enzyme (36). The presence of anti-regucalcin monoclonal antibody (25 or 50 ng/ml) in the enzyme reaction mixture led to a significant increase in NO synthase activity and this effect was completely abolished by the addition of regucalcin. Therefore, endogenous regucalcin in the heart cytoplasm may act as a suppressor protein in the regulation of NO synthase activity.

Of note, NO synthase activity was not altered in the heart muscle cytoplasm obtained from regucalcin TG rats, which overexpress endogenous regucalcin compared to wild-type rats (36). However, the stimulatory effect of Ca^2+^ (10 μM) addition on NO synthase activity was weakened in the heart muscle cytoplasm obtained from regucalcin TG rats (36). This finding supports the hypothesis that endogenous regucalcin may exert a suppressive effect on NO synthase activity in the heart muscle cytoplasm of rats.

The physiological significance of regucalcin inhibition on NO synthase in heart muscle cytoplasm is unknown. However, regucalcin may participate in the regulation of NO production in heart muscle cells. NO acts as a messenger or modulator molecule in heart muscle. NO production may be stimulated through Ca^2+^ signaling due to hormonal stimulation in heart muscle cells. Regucalcin may exert a suppressive effect on the overproduction of NO due to inhibiting NO synthase in heart muscle cells.

## 5. Other role of regucalcin in heart cell regulation

Superoxide dismutase (SOD) plays a role in the prevention of cell death and apoptosis in the heart (37). A decrease in manganese SOD activity is associated with increased mitochondrial oxidative damage, as demonstrated by the decrease in the activities of iron SH proteins sensitive to oxygen stress (37). Cu/Zn-SOD was shown to act as a protector against dexorubicin-induced cardiotoxicity in mice (38). Furthermore, NO is involved in the control of myocardial O_2_ consumption in rats (39). Regucalcin was found to increase SOD activity in rat heart cytoplasm (40).

The addition of regucalcin (10^−10^-10^−8^ M) at a physiological concentration to the enzyme reaction mixture containing the heart cytoplasm obtained from wild-type rats led to an increase in SOD activity, indicating that regucalcin directly activates this enzyme (40). The stimulatory effect of regucalcin on SOD activity was not observed in the presence of DTT, a protecting reagent for SH groups, or NEM, a modifying reagent for SH groups, in the reaction mixture, indicating that regucalcin does not affect the SH groups (40). The addition of zinc sulfate to the reaction mixture did not lead to a significant change in SOD activity, whereas the enzyme activity was markedly decreased in the presence of cupric sulfate (40). The activating effect of regucalcin on SOD was observed in the presence of zinc, whereas it was not observed in the presence of copper (40). Moreover, SOD activity was enhanced in the heart cytoplasm of regucalcin TG rats compared to the wild-type rats (40). The abovementioned findings demonstrate that regucalcin increases SOD activity in the heart cytosol of rats and this effect is not associated with the enzyme SH groups.

Regucalcin was found to increase SOD activity in rat heart cytoplasm. Regucalcin exerts an inhibitory effect on NO synthase activity in the heart cytosol (36). The production of superoxide radicals is known to be the cause of cardiac damage. Regucalcin may participate in the regulation of the production of superoxide radicals in rat heart muscle cells.

Ageing is an important risk factor of cardiovascular diseases, including heart failure. The role of regucalcin in cardiac remodelling was previously reported (41). Regucalcin-knockout and wild-type mice were subjected to continuous angiotensin II infusion. This treatment caused more prominent cardiac hypertrophy and myocardial fibrosis in regucalcin-knockout compared to those observed in wild-type mice (41). Regucalcin-knockout mice exhibited increased generation of reactive oxygen species, increased number of deoxynucleotidyl transferase-mediated dUTP nick end-labelling positive nuclei, activation of caspase-3, increases in the BAX:Bcl-2 ratio and phosphorylation of c-Jun N-terminal kinase (41). Thus, regucalcin deficiency may exacerbate angiotensin II-induced cardiac hypertrophy, dysfunction and remodelling. Regucalcin may play a cardioprotective role in cardiac remodelling in response to angiotensin II, due to its antioxidative and anti-apoptotic properties.

## 6. Prospect

Regucalcin plays a pivotal role as a suppressor protein in Ca^2+^-related signal transduction in various types of cells and tissues, including the liver and kidney (10–12). Moreover, regucalcin was demonstrated to regulate intracellular Ca^2+^ homeostasis due to activating the Ca^2+^-ATPase in the sarcoplasmic reticulum and mitochondria of rat heart cells. Regucalcin suppresses Ca^2+^/calmodulin-dependent enzymes, including protein phosphatase and NO synthase, which are associated with Ca^2+^ signaling. Ca^2+^ signaling is one of the most important components in cardiac excitation-contraction coupling. This coupling system may be regulated through regucalcin. Regucalcin may play a physiological role in the regulation of Ca^2+^-related heart cell functions. Whether regucalcin is associated with other protein molecules that are involved in cardiac excitation-contraction coupling in heart cells, remains to be elucidated.

Moreover, regucalcin was found to play a pivotal role as a suppressor of NO synthase and an activator of SOD in the heart cytoplasm. The overproduction of NO may lead to heart cell damage. SOD plays a pivotal role in the suppression of free radical production that leads to heart failure. Regucalcin may play a physiological role by exerting protective effects against heart failure, through the activation of SOD or the suppression of NO overproduction in heart cells. The pathophysiological role of regucalcin in heart dysfunction remains to be fully elucidated. However, the currently available evidence indicate that regucalcin may be a target molecule in heart disease.
